# The arrhythmogenic cardiomyopathy phenotype associated with *PKP2* c.1211dup variant

**DOI:** 10.1007/s12471-023-01791-2

**Published:** 2023-07-28

**Authors:** Thomas A. Bos, Sebastiaan R. D. Piers, Marja W. Wessels, Arjan C. Houweling, Regina Bökenkamp, Marianne Bootsma, Laurens P. Bosman, Reinder Evertz, Debby M. E. I. Hellebrekers, Yvonne M. Hoedemaekers, Jeroen Knijnenburg, Ronald Lekanne Deprez, Anneke M. van Mil, Anneline S. J. M. te Riele, Marjon A. van Slegtenhorst, Arthur A. M. Wilde, Sing-Chien Yap, Dennis Dooijes, Tamara T. Koopmann, J. Peter van Tintelen, Daniela Q. C. M. Barge-Schaapveld, European Reference Network for rare, low prevalence and complex diseases of the heart: ERN GUARD-Heart, Arjan C. Houweling, Ronald Lekanne Deprez, Anneline S. J. M. te Riele, Arthur A. M. Wilde, J. Peter van Tintelen

**Affiliations:** 1 Department of Clinical Genetics, Leiden University Medical Centre, Leiden, The Netherlands; 2 Department of Cardiology, Leiden University Medical Centre, Leiden, The Netherlands; 3 Department of Clinical Genetics, Erasmus University Medical Centre, Rotterdam, The Netherlands; 4 Department of Human Genetics, Amsterdam University Medical Centres, Amsterdam, The Netherlands; 5 Department of Paediatric Cardiology, Leiden University Medical Centre, Leiden, The Netherlands; 6 Netherlands ACM Registry, Utrecht, The Netherlands; 7 Department of Cardiology, Radboud University Medical Centre, Nijmegen, The Netherlands; 8 Department of Clinical Genetics, Maastricht University Medical Centre, Maastricht, The Netherlands; 9 Department of Human Genetics, Radboud University Medical Centre, Nijmegen, The Netherlands; 10 Department of Heart and Lungs, Division of Cardiology, University Medical Centre Utrecht, Utrecht, The Netherlands; 11 Heart Centre, Department of Cardiology, Amsterdam Cardiovascular Sciences, Heart Failure and Arrhythmias, Amsterdam University Medical Centres, Amsterdam, The Netherlands; 12 Department of Cardiology, Erasmus University Medical Centre, Rotterdam, The Netherlands; 13 Department of Clinical Genetics, University Medical Centre Utrecht, Utrecht, The Netherlands

**Keywords:** Arrhythmogenic Cardiomyopathy, Plakophilin‑2, Founder mutation, Genetics

## Abstract

**Background:**

The arrhythmogenic cardiomyopathy (ACM) phenotype, with life-threatening ventricular arrhythmias and heart failure, varies according to genetic aetiology. We aimed to characterise the phenotype associated with the variant c.1211dup (p.Val406Serfs*4) in the plakophilin‑2 gene (*PKP2*) and compare it with previously reported Dutch *PKP2* founder variants.

**Methods:**

Clinical data were collected retrospectively from medical records of 106 *PKP2* c.1211dup heterozygous carriers. Using data from the Netherlands ACM Registry, c.1211dup was compared with 3 other truncating *PKP2* variants (c.235C > T (p.Arg79*), c.397C > T (p.Gln133*) and c.2489+1G > A (p.?)).

**Results:**

Of the 106 carriers, 47 (44%) were diagnosed with ACM, at a mean age of 41 years. By the end of follow-up, 29 (27%) had experienced sustained ventricular arrhythmias and 12 (11%) had developed heart failure, with male carriers showing significantly higher risks than females on these endpoints (*p* < 0.05). Based on available cardiac magnetic resonance imaging and echocardiographic data, 46% of the carriers showed either right ventricular dilatation and/or dysfunction, whereas a substantial minority (37%) had some form of left ventricular involvement. Both geographical distribution of carriers and haplotype analysis suggested *PKP2* c.1211dup to be a founder variant originating from the South-Western coast of the Netherlands. Finally, a Cox proportional hazards model suggested significant differences in ventricular arrhythmia–free survival between 4 *PKP2* founder variants, including c.1211dup.

**Conclusions:**

The *PKP2* c.1211dup variant is a Dutch founder variant associated with a typical right-dominant ACM phenotype, but also left ventricular involvement, and a possibly more severe phenotype than other Dutch *PKP2* founder variants.

**Supplementary Information:**

The online version of this article (10.1007/s12471-023-01791-2) contains supplementary material, which is available to authorized users.

## What’s new?

- The *PKP2* c.1211dup variant is a Dutch founder variant that is associated with a typical right-sided arrhythmogenic cardiomyopathy phenotype and also milder but substantial left-sided involvement.
- Ventricular arrhythmia (VA) was an early, predominant manifestation, occurring in nearly a third of carriers from 14 years of age onwards. Heart failure, on the other hand, was uncommon before the age of 55 years.
- VA occurred earlier in life in male carriers compared with female carriers.
- About 60% of carriers remained asymptomatic by age 60.
- Dutch *PKP2* truncating founder variants may differ in phenotype severity.

## Introduction

Arrhythmogenic cardiomyopathy (ACM) is an umbrella term describing a range of progressive, often familial cardiomyopathies resulting in ventricular arrhythmia (VA) and ventricular dilatation [1–3]. The best characterised subtype of ACM is arrhythmogenic right ventricular cardiomyopathy (ARVC), which is diagnosed according to the 2010 Revised Task Force Criteria [4]. ARVC patients can show considerable clinical variation [5], in part due to age-related penetrance and risk factors such as male sex, frequent endurance exercise and genetic aetiology [6–8].

The most commonly identified causal gene in patients with ARVC is plakophilin‑2 (*PKP2*), with pathogenic variants found in nearly half of a series of 439 Dutch and American probands with ARVC [9]. *PKP2* variant carriers typically exhibit a right ventricular (RV) dominant disease progression. Although left ventricular (LV) involvement has been acknowledged increasingly for desmosomal variants in general, the evidence still indicates less common involvement of *PKP2* variants [10].

The heterozygous truncating *PKP2* variant, c.1211dup (p.Val406Serfs*4), alternatively referred to as c.1212insT, was initially reported in a cohort of 56 patients fulfilling the 1994 ARVC Task Force Criteria [11] and regularly since then (ClinVar Variation ID 45015). More recently, homozygosity of *PKP2* c.1211dup has been associated with a severe form of hypoplastic left heart syndrome [12]. Still, no large cohort of individuals carrying the c.1211dup variant of the *PKP2* gene has been studied. As genetic factors likely contribute to facets of the ACM phenotype, such as onset of heart failure (HF) and degree of LV involvement [8], and considering that treatment decisions (pharmacotherapy and/or device therapy) depend on phenotype severity [3], genotype-phenotype research is essential to adequately inform patients and physicians and guide them in the decision-making process.

**Fig. 1:**
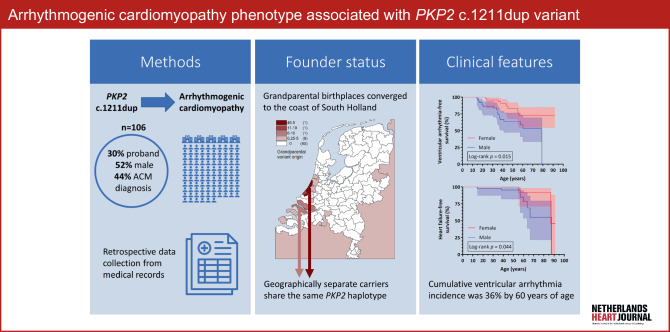
Infographic

In the current report, we describe the phenotype of the *PKP2* c.1211dup variant (Fig. 1) and compare it with other Dutch *PKP2* founder variants.

## Methods

### Study population

Of the 123 *PKP2* c.1211dup heterozygous carriers identified through the academic DNA diagnostic laboratories in the Netherlands, 2 refused informed consent and 15 had no follow-up medical records. Obligate carriers were not included due to lack of systematic cardiological evaluation. In addition to the heterozygous carriers, 2 patients homozygous for the *PKP2* c.1211dup variant were identified and excluded.

All VA endpoints and baseline characteristics of carriers of 3 other *PKP2* variants (c.235C > T (p.Arg79*), *n* = 53; c.397C > T (p.Gln133*), *n* = 42; and c.2489+1G > A (p.?), *n* = 18) were exported from the Netherlands ACM Registry and compared with that of *PKP2* c.1211 dup carriers from the Netherlands ACM Registry (*n* = 46) using VA-free survival.

Apart from one male proband who presented with hypertrophic cardiomyopathy (next generation sequencing panel for cardiomyopathy genes revealed no additional (likely) pathogenic variant), no other type of cardiomyopathy than ARVC was observed.

All participants provided informed consent, according to the local medical ethics committees of all participating medical centres and/or conform the requirements of the Netherlands ACM Registry [13]. The study was conducted in accordance with the principles of the Declaration of Helsinki.

### Data collection

In addition to the information required to assess diagnostic status according to the 2010 Revised Task Force Criteria, we initially collected data on medical history, medication use and exercise history from the Netherlands ACM Registry (*n* = 46) [13]. Electronic medical records of all participating medical centres were used to corroborate and supplement these data where possible (see Table S1 in Electronic Supplementary Material). In case of any discrepancies, data from primary medical records were used. Data collection for the remaining 60 carriers, not included in the ACM Registry, was based on the electronic medical records of the participating centres.

Sustained VA was defined as a composite of sudden cardiac death (SCD), sudden cardiac arrest, spontaneous sustained ventricular tachycardia (VT) (≥ 30 s at ≥ 100 bpm or with haemodynamic compromise requiring cardioversion), ventricular fibrillation/flutter (VF) or appropriate implantable cardioverter-defibrillator (ICD) intervention. This definition was also used for development of the ARVC risk calculator [14]. HF was defined by cardiological diagnosis with symptoms graded as New York Heart Association class ≥ 2 (for all definitions, see Table S2 in Electronic Supplementary Material).

### Genetic evaluation

All participants were genetically tested between June 2005 and July 2021. Probands were tested conform standard practices at the time of genetic testing, whereas family members were usually only tested for family variants (see Table S3 in Electronic Supplementary Material). Genetic testing revealed no other (likely) pathogenic variants in any participant (see Table S4 in Electronic Supplementary Material).

Pedigrees constructed from patient records, government archives and online genealogical records were used to find a common ancestor (see ‘Pedigree evaluation’ in Supplemental Methods in Electronic Supplementary Material). Participant and grandparent birthplaces were generalised to 2‑digit postal codes. Geographical distribution maps were generated with MapInfo Pro 2019 (Precisely, Burlington, MA, USA).

Haplotype analysis using the CytoScan HD single nucleotide polymorphism (SNP) array was performed according to the manufacturer’s instructions on 4 samples from two different, geographically separated pedigrees carrying the *PKP2* c.1211dup variant for which no common ancestor could be found (see ‘Haplotype analysis’ in Supplemental Methods in Electronic Supplementary Material).

### Statistical analysis

The statistical analyses are described in detail in the Supplemental Methods. Two-tailed *p*-values < 0.05 were interpreted as statistically significant.

## Results

### Cohort characteristics

In total, 106 heterozygous *PKP2* c.1211dup carriers (30% proband, 52% male, mean age at genetic testing 43 ± 20 years) were included (Tab. 1). The most common symptom at presentation for all carriers was sustained VT or VF (20%). Fewer carriers (12%) reported syncope. The median follow-up duration was 3.4 years (IQR: 0.4–10.8). Carriers were treated primarily with beta-blockers (45%) and/or underwent an ICD implantation (33%).

**Table 1:** Summary of *PKP2* c.1211dup proband and family member characteristics

Variable	Probands (*n* = 32)	Family members (*n* = 74)
*Demographics*		
Male	19 (59)	36 (49)
Age at presentation, years	43 ± 16	43 ± 21
Follow-up duration, years	5.4 (1.1–12.2)	3.0 (0.3–8.7)
Age at diagnosis, years	39 ± 17	46 ± 21 (*n* = 15)
*At presentation, n (%)*		
*2010 Revised Task Force Criteria*		
Structural alterations (major)	24 (75)	2 (3)
Structural alterations (minor)	0	7 (10)
Tissue characterisation (major)	0	0
Tissue characterisation (minor)	0	0
Repolarisation abnormalities (major)	19 (59)	4 (5)
Repolarisation abnormalities (minor)	6 (19)	5 (7)
Depolarisation abnormalities (major)	4 (13)	0
Depolarisation abnormalities (minor)	9 (28)	8 (11)
Arrhythmia (major)	16 (50)	4 (5)
Arrhythmia (minor)	12 (38)	7 (10)
*Risk factors*		
Endurance sport	13/21 (62)	18/39 (46)
Hypertension	4/25 (16)	21/58 (39)
(Ex‑)smoker	4/18 (22)	15/45 (33)
Dyslipidaemia	4/24 (17)	14/48 (29)
*Symptoms*	*n* = 31	*n* = 68
Syncope	7 (23)	5 (7)
VT/VF	18 (58)	2 (3)
Sudden cardiac arrest	4 (13)	1/67 (1)
NYHA class ≥ 2	2 (6)	1 (1)
Supraventricular tachycardia	7 (23)	9 (13)
*By end of follow-up, n (%)*		
*Invasive treatment modalities*	*n* = 31	*n* = 69
ICD implantation	22 (69)	11 (16)
Heart transplantation	1 (3)	0
Ablation (VT)	11 (34)	0
Ablation (other indications)	7 (21)	2 (3)
*Medication*	*n* = 31	*n* = 65
Beta-blockers^a^	24 (77)	19 (29)
Antiarrhythmics (Class I)	3 (10)	2 (3)
Antiarrhythmics (Class III)^a^	17 (55)	5 (8)
Mineral receptor antagonists	6 (19)	1 (2)
ARNIs	2 (6)	1 (2)
ACEI/ARBs	6 (19)	13 (20)
Diuretics	6 (19)	8 (12)

Data are *n* (%), mean ± standard deviation, or median (interquartile range)

*VT* ventricular tachycardia, *VF* ventricular fibrillation, *NYHA* New York Heart Association, *ICD* implantable cardioverter-defibrillator, *ARNI* angiotensin receptor–neprilysin inhibitor, *ACEI* angiotensin converting enzyme inhibitor, *ARB* angiotensin-receptor blocker

^a^ Sotalol was classified as both a beta-blocker and class III antiarrhythmic agent

### ACM phenotype

#### Disease onset

**Fig. 2:**
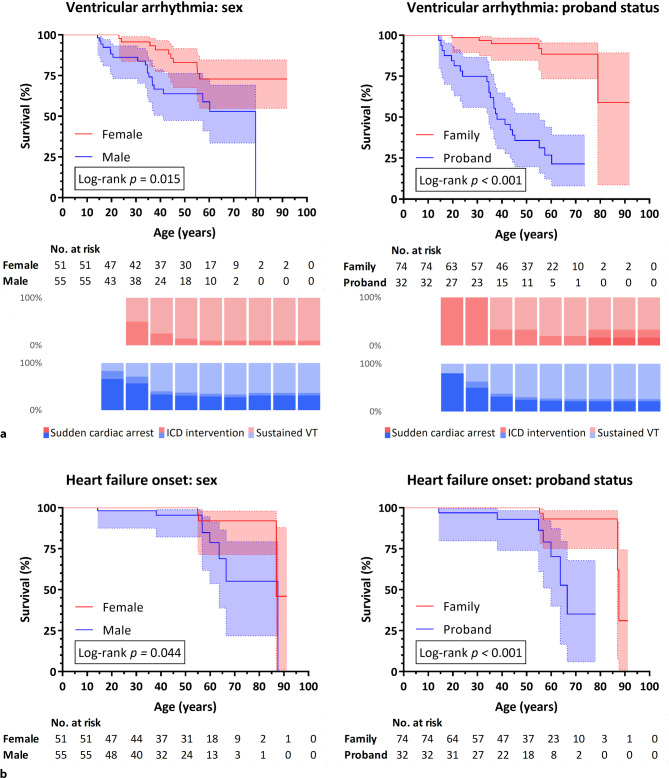
Kaplan-Meier survival analyses of onset of **a** ventricular arrhythmia and **b** heart failure. Survival curves were stratified by sex (*left*) and proband status (*right*). For definitions, see Table S2 in Electronic Supplementary Material. Error bands represent 95% confidence intervals. Number at risk (carriers who did not yet experience an event or censoring) is indicated in 10-year intervals. Cumulative distribution of ventricular arrhythmia events are indicated in 10-year intervals underneath survival curves in panel a (*ICD* implantable cardioverter-defibrillator, *VT* ventricular tachycardia)

Overall, 47 carriers (44%) fulfilled the ARVC diagnostic criteria, at a mean age of 41 ± 19 years (range: 12–87), with no significant differences by proband status (*p* = 0.24) or sex (*p* = 0.17). However, sustained VA and HF was diagnosed significantly earlier in males than females (Fig. 2). By the age of 40 years, 33% of men (versus 9% of women) had experienced sustained VA. HF occurred at a later age than sustained VA for both men (5% by age 40 years, increasing to 21% at age 60 years) and women (no HF by age 40 years, increasing to 8% at age 60 years). Both events also occurred significantly earlier in probands than relatives (Fig. 2). Notably, by the age of 40 years, only 5% of relatives had experienced sustained VA and none had developed HF.

Moreover, 7 male carriers (5 of whom were ≤ 35 years old) experienced sudden cardiac arrest, whereas only 1 female carrier experienced sudden cardiac arrest (at age 65 years). One 17-year-old male underwent heart transplantation due to HF. In total, 4 deaths (3 men and 1 woman) were observed following end-stage HF. Of note, 6 men with a 50% chance of carrying the c.1211dup variant experienced SCD, between 16 and 70 years old.

#### Phenotypic features

VA was a predominant disease manifestation, with 29 carriers (27%) experiencing sustained VA (mean age: 37 ± 16 years) and only 12 carriers (11%) experiencing HF (mean age: 58 ± 20 years). In addition, 34% (probands 72%, family members 16%) of carriers with available ambulatory electrocardiographic monitoring had a premature ventricular contraction (PVC) burden > 1%. Median PVC burden in this subgroup was 2.6% (IQR 1.8–6.4%).

RV dilatation and dysfunction was found in 40 (45%) and 43 (46%) carriers, respectively, by cardiac magnetic resonance (CMR) imaging and/or echocardiography, occurring more frequently in probands than their relatives (Tab. 2). Of the 93 carriers with available CMR images or echocardiograms, 20 (22%) showed LV dilatation, whereas 34 carriers (37%) exhibited LV dysfunction. Additionally, 12 of the 36 carriers (33%) with appropriate images showed LV late gadolinium enhancement.

**Table 2:** Left and right ventricular involvement on cardiac imaging^a^

Variable	Probands	Family members	*P*-value
*CMR*^*b*^			
RVEDVi, ml/m^2^	146 ± 37	96 ± 18	
RVEF, %	37 ± 12	53 ± 7.6	
LVEDVi, ml/m^2^	99 ± 18	89 ± 17	
LVEF, %	54 ± 15	59 ± 7.2	
*CMR*			
RV dilatation	19/20 (95)	11/29 (38)	< 0.001
RV dysfunction	16/18 (89)	9/28 (32)	< 0.001
LV dilatation	10/20 (50)	6/29 (21)	0.032
LV dysfunction	10/21 (48)	10/29 (34)	0.349
LV LGE	7/15 (47)	5/21 (24)	0.151
*Echocardiography*			
RV dilatation	20/27 (74)	7/58 (12)	< 0.001
RV dysfunction	21/28 (75)	8/61 (13)	< 0.001
LV dilatation	5/27 (19)	0/59	0.002
LV dysfunction	15/30 (50)	6/61 (10)	< 0.001

Data are mean ± standard deviation or number affected/total number available (%)

*EDVi* indexed end-diastolic volume, *EF* ejection fraction, *LGE* late gadolinium enhancement

^a^ Ventricular imaging parameters on most recent cardiac magnetic resonance (*CMR*) image or 2D-echocardiogram. Dilatation was defined as any indexed end-diastolic volume > 1.96 standard deviations above reference population mean on CMR image and as determined by eyeballing on echocardiogram. Dysfunction was defined as any ejection fraction > 1.96 standard deviations below reference population mean on CMR image and as determined by eyeballing on echocardiogram

^b^ *n* = total number available (provided in CMR data on dilatation and dysfunction of right ventricle (*RV*) and left ventricle (*LV*))

### Founder status

#### Geographical distribution and pedigree evaluation

**Fig. 3:**
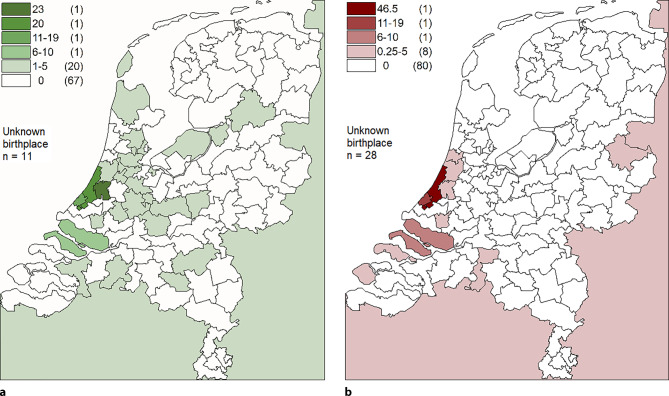
Origin of *PKP2* c.1211dup variant in the Netherlands, divided in 2‑digit postal code regions. Numbers of **a** carriers and **b** grandparents with c.1211dup variant are shown. If unknown which grandparent carried *PKP2* c.1211dup, multiple grandparents were weighted. Numbers between parentheses represent total number of regions per range. Land area outside of the Netherlands represents all individuals born outside of the Netherlands

The majority of *PKP2* c.1211dup carriers were born in the South-West of the Netherlands (Fig. 3). Grandparental birthplaces converged to 3 coastal communities in the province of South Holland. Pedigrees from 2 communities (13/29) could be linked to a putative common ancestral couple (range: 8–11 generations), which was born in the late 17th century (see Figure S1 in Electronic Supplementary Material).

#### Haplotype analysis

CytoScan HD SNP array values on chromosome 12 of 2 individuals from the genealogically linked communities and 2 individuals from the third community were compared (see Supplemental Dataset S1 and Supplemental Results in Electronic Supplementary Material). All individuals showed a matching 1.2-Mb region encompassing the *PKP2* gene on ≥ 1 allele.

### Variant-specific phenotypic variability

The 4 *PKP2* variant groups (c.235C > T, c.397C > T, c.1211dup and c.2489+1G > A) are described in Table S5 in Electronic Supplementary Material. A Cox proportional hazards model adjusted for sex and proband status showed c.1211dup to be associated with a significantly higher rate of sustained VA compared with c.235C > T but not compared with c.397C > T or c.2489+1G > A (Tab. 3).

**Table 3:** Ventricular arrhythmia–free survival among *PKP2* founder variants^a^

Variable	Coefficient (SE)	HR (95% CI)	*P*-value
*Sex*			
Female	Ref		
Male	0.494 (0.274)	1.64 (0.968–2.85)	0.072
*Proband status*			
Family member	Ref		
Proband	2.37 (0.274)	10.7 (5.87–20.8)	< 0.001
PKP2* variant*			
c.235C > T	−0.717 (0.338)	0.489 (0.249–0.947)	0.034
c.397C > T	−0.546 (0.362)	0.579 (0.278–1.17)	0.131
c.1211dup	Ref		
c.2489+1G > A	0.737 (0.386)	2.09 (0.95–4.39)	0.056

*SE* standard error, *HR* hazard ratio, *CI* confidence interval, *Ref* reference group

^a^ Comparison of *PKP2* c.1211dup with 3 other *PKP2* founder variants assessed by using Cox proportional hazards model adjusted for sex and proband status

## Discussion

Our study has provided a detailed characterisation of the ACM phenotype of over 85% of all known Dutch heterozygous carriers of the *PKP2* c.1211dup variant. In agreement with previously described *PKP2* variants [2], the ACM phenotype associated with the *PKP2* c.1211dup variant showed incomplete penetrance and variable expression and was skewed towards male carriers. VA was an early, predominant manifestation in c.1211dup carriers, occurring mostly before the age of 40 but documented as early as 14 years old. Additionally, 33% had a high PVC burden. HF, on the other hand, was uncommon before the age of 55 years. In three studies documenting 434 *PKP2* variant carriers [8], 53 *PKP2* c.235C > T carriers [15] and 14 *PKP2* c.2489+4A > C carriers [16], respectively, VA was also a predominant disease manifestation, whereas HF occurred in ≤ 3%. Although the higher incidence of HF currently found might be specifically related to the c.1211dup variant, a more likely explanation is the large number of carriers (34%) who were older than 60 years by the end of follow-up.

As could be expected from other *PKP2* studies, the c.1211dup variant was associated with a RV-dominant phenotype. However, our study adds to the growing body of evidence that desmosomal variants, even in *PKP2,* may be related to a significant degree of LV involvement [8, 17, 18]. In the entire cohort, at least 19% (20/106) showed LV dilatation and 32% (34/106) LV dysfunction on CMR imaging and/or echocardiography. The degree of LV dilatation and dysfunction was generally mild, with only 12 carriers (11%) showing symptomatic HF. Any discrepancies between CMR and echocardiographic data are most likely explained by echocardiography’s lower sensitivity [19].

Geographic data and the putative common ancestral couple in the late 17th century strongly suggested that in the Netherlands, the *PKP2* c.1211dup variant originated from one of the historic fishing villages on the coast of the province of South-Holland. Contrary to an earlier report on 2 haplotypes [20], we found an identical haplotype sharing a 1.2-Mb stretch surrounding *PKP2* based on 4 carriers from two geographically distinct populations. Although we cannot fully exclude a second haplotype, a recombination event can also not be excluded in the previous study, as the 2 previously identified haplotypes overlapped on ≥ 140 kb. Based on current data, *PKP2* c.1211dup can be regarded as one of several *PKP2* founder variants prevalent in the Netherlands [15, 20]. Despite the relatively high regional frequency of this variant, no additional cases of homozygosity, which were previously shown to be associated with hypoplastic left heart syndrome [12], were observed.

Based on our results, the current recommendations on clinical management and family screening—with cardiological screening from the age of 10 onwards and no upper age limit in patients and family members with the *PKP2* c.1211dup variant—is justified [3]. Currently, risk calculators only exist for patients recently fulfilling diagnostic criteria [14]. Further research is warranted on risk stratification of carriers who showed relatively low risks of VA and HF.

### Study limitations

In addition to the inherent limitations of our study’s retrospective design (such as limited data on cardiac inflammation and exercise history, which are both of pathophysiological interest [7, 24]), the current results should be interpreted in the context of the following considerations. Firstly, we cannot rule out that the 17 carriers who could not be included may have had a lower disease burden, possibly leading to an overestimation of disease severity in this study. On the other hand, carriers were not followed-up from birth, which could have led to underestimation of phenotype severity in the form of ascertainment bias of carriers surviving long enough to undergo genetic testing. Another potential source of ascertainment bias is presented by 6 men with a 50% chance of carrying *PKP2* c.1211dup who suffered SCD between the ages of 16 and 70 years old but could not be included in our cohort due to lack of genetic test results (data not shown).

Furthermore, the unexpected difference found in VA risk between 4 truncating *PKP2* variants suggested that *PKP2* truncating variant carriers may carry different risks depending on variant location. However, our approach was limited in sample size and available clinical data, only allowing us to compare the founder variants on VA-free survival. Additionally, mechanistic work on* PKP2* truncating variants so far has generally suggested a haploinsufficiency mechanism [21–23], which would exclude variant-specific influences on phenotype severity. Our results should therefore be interpreted with caution and corroborated in more elaborate studies. Phenotype differences may also be explained by unknown coinherited factors or environmental influences.

## Conclusion

Our results confirm the *PKP2* c.1211dup variant to be a Dutch founder variant associated with a typical right-sided ACM phenotype and also milder but substantial left-sided involvement. Although penetrance at the age of 60 years was only 40%, sustained VA mostly occurred prior to the age of 40 years and may present from 14 years of age onwards, with an increased risk for males. In contrast, HF was less common, particularly prior to the age of 55 years. Further studies including more clinical data should confirm whether phenotype severity is dependent on the variant location in *PKP2* truncating variants.

## Supplementary Information

**Supplemental Methods**

**Supplemental Results**

**Figure S1** Dendrogram of relationship of probands to putative ancestral couple

**Table S1** Number of participants with available data per diagnostic test

**Table S2** Definitions

**Table S3** Genetic sequencing techniques and genes tested

**Table S4** Other class ≥ 3 genetic variants in *PKP2* c.1211dup carriers at time of genetic testing

**Table S5** Summary of *PKP2* founder variant characteristics

**Supplemental Dataset S1** SNP analysis
