# Supplementary material for: The arrhythmogenic cardiomyopathy phenotype associated with PKP2 c.1211dup variant
Source: Neth Heart J. 2023 Jul 28;31(7-8):315–23. doi: 10.1007/s12471-023-01791-2 (PMC10400759; doi:10.1007/s12471-023-01791-2)
Supplement: Supplementary file 1 — Supplemental Methods [file 12471_2023_1791_MOESM1_ESM.docx]

**Supplemental Methods**

*Data sources*

Data collection forms and database were designed using Castor EDC v2021.4.5 (Castor, Amsterdam, The Netherlands). Data minimisation, encryption strategy and database design were externally validated prior to data collection. Data was collected between June 2021 and January 2022. Data was sourced from existing electronical medical records and archives of all participating medical centres.

*Pedigree evaluation*

Pedigrees were traced genealogically and geographically. Family trees were constructed from patient records, government archives and online genealogical records. Pedigrees were traced for six generations and compared to find common ancestors. Frequently overlapping ancestors were then selected and traced for further generations, until a common ancestral couple was found. In cases of multiple links to the ancestral couple, the lineage with the greatest mortality was assumed to carry the mutation. Mortality was defined as ratio of age of death to age of death of spouse.

*Haplotype analysis*

Haplotype analysis using CytoScan HD single nucleotide polymorphism (SNP) array was performed according to manufacturer’s instructions on four samples from two different, geographically separated pedigrees carrying the PKP2 c.1211dup variant for which no common ancestor could be found. The resulting SNP values of the four individuals of the included SNPs on chromosome 12 were compared in order to determine genomic regions with matching, concordant allelic SNP values for at least one allele between all four compared individuals. As a comparison, a control experiment using SNP values of four separate unrelated samples, investigated for other reasons, was compared with each other in the exact same way.

*Statistical analysis*

Categorical variables are expressed as number (percentage), and continuous variables as mean ± standard deviation or median (interquartile range (IQR)). Categorical variables were compared using the χ2 test or the Fisher exact test. Continuous variables were compared using the Mann-Whitney U test or the Student’s T test, when appropriate.

Kaplan-Meier curves were censored at end of follow-up or upon death due to non-cardiac causes and were compared by log-rank tests. A Cox proportional hazards model, corrected for sex and proband status was used to compare ventricular arrhythmia-free survival between *PKP2* c.1211dup and three other *PKP2* founder variants. Proportional hazards assumptions were tested using log-minus-log survival plots and Schoenfeld residuals against time. Kaplan-Meier curves, the Cox proportional hazard model, 95% confidence intervals and log-rank test results were generated using Graphpad Prism v9.2.0 (GraphPad Software, California, USA). All other statistics were performed using IBM SPSS Statistics v29.0.0.0 (IBM, New York, USA).
