# Supplementary material for: The arrhythmogenic cardiomyopathy phenotype associated with PKP2 c.1211dup variant
Source: Neth Heart J. 2023 Jul 28;31(7-8):315–23. doi: 10.1007/s12471-023-01791-2 (PMC10400759; doi:10.1007/s12471-023-01791-2)
Supplement: Supplementary file 2 — Supplemental Results [file 12471_2023_1791_MOESM2_ESM.docx]

**Supplemental Results**

*Haplotype analysis*

CytoScan HD SNP array values on chromosome 12 of four selected individuals from the two geographically separated pedigrees were compared (see Supplemental data set S1 – SNP analysis). In this comparison, in addition to two other matching regions, a stretch of 1.2 MB containing 337 consecutive SNPs was found on chromosome 12 (32.186.868 bp to 33.369.315 bp) in which all 337 SNPs in these four individuals shared at least one allele. This region encompasses the *PKP2* gene (32.943.680-33.049.780 bp) completely (all genomic positions are in GRCh37). In a control experiment using four unrelated samples, investigated for other reasons, no stretch of a homologous allele in this region of chromosome 12 was found. These comparisons provide support for a shared haplotype, supporting a common ancestor (prior to the late 17^th^ century).
