# Supplementary material for: The arrhythmogenic cardiomyopathy phenotype associated with PKP2 c.1211dup variant
Source: Neth Heart J. 2023 Jul 28;31(7-8):315–23. doi: 10.1007/s12471-023-01791-2 (PMC10400759; doi:10.1007/s12471-023-01791-2)
Supplement: Supplementary file 3 — Figure S1 Dendrogram of relationship of probands to putative ancestral couple [file 12471_2023_1791_MOESM3_ESM.docx]

**
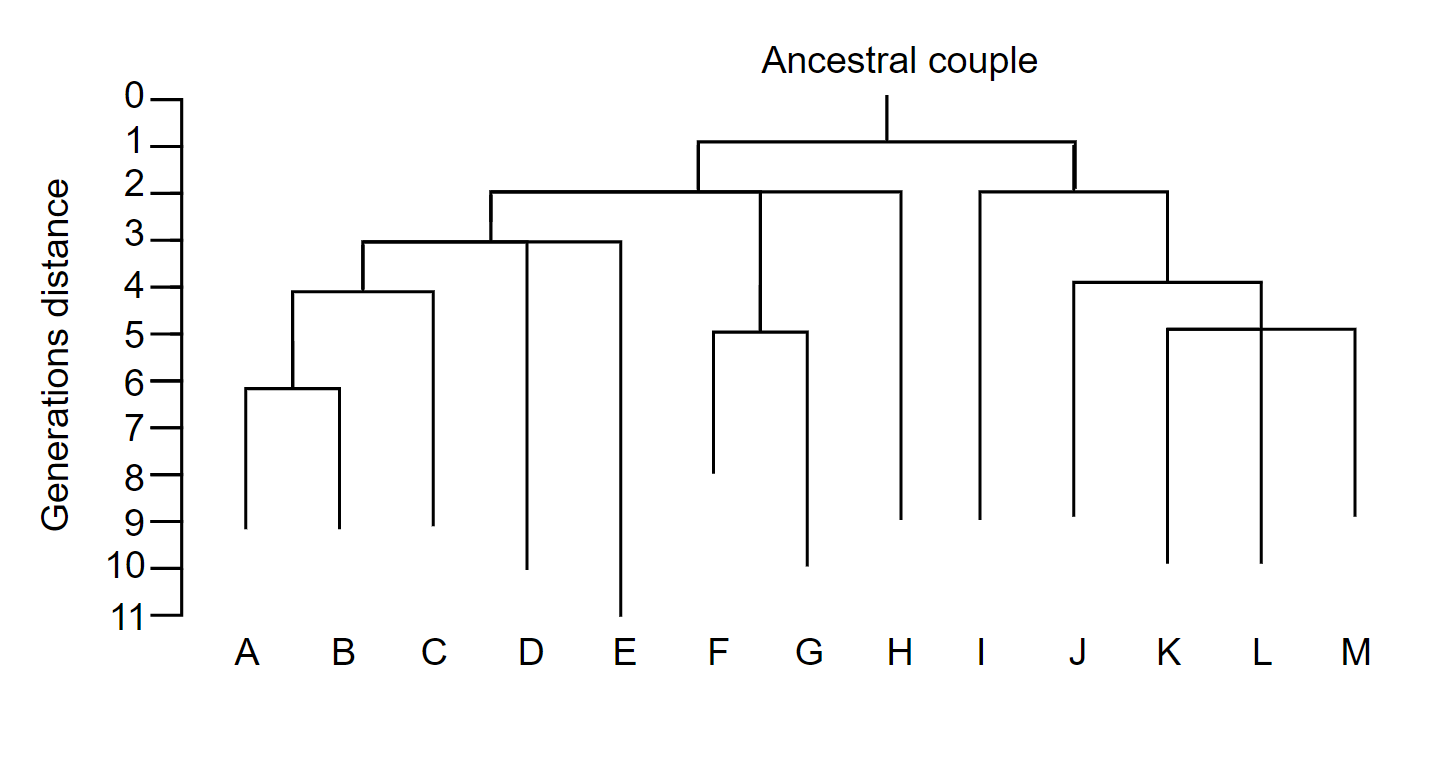
Figure S1** Dendrogram of relationship of probands to putative ancestral couple

*Thirteen probands, labelled A-M, are clustered by genealogical distance from one another. Probands E, I, J, K, L and M were related via more than one path. In these cases, the median mortality of all ancestors linking a proband to the putative ancestral couple was used to determine the most likely path of mutation transmission.*
