# Supplementary material for: The arrhythmogenic cardiomyopathy phenotype associated with PKP2 c.1211dup variant
Source: Neth Heart J. 2023 Jul 28;31(7-8):315–23. doi: 10.1007/s12471-023-01791-2 (PMC10400759; doi:10.1007/s12471-023-01791-2)
Supplement: Supplementary file 4 — Table S1 Number of participants with available data per diagnostic test [file 12471_2023_1791_MOESM4_ESM.docx]

**Table S1** Number of participants with available data per diagnostic test

| **n (%)** | **Total availability (n=106)** | **Proband availability (n=32)** | **Family member availability (n=74)** |
| --- | --- | --- | --- |
| 2D-Echocardiography* | 92 (87) | 30 (94) | 62 (84) |
| CMR^*^ | 54 (51) | 25 (78) | 29 (39) |
| Angiography | 27 (25) | 19 (59) | 8 (11) |
| 12-lead ECG | 91 (86) | 31 (97) | 60 (81) |
| Ambulatory ECG | 79 (75) | 25 (78) | 54 (73) |
| Exercise ECG | 77 (73) | 26 (81) | 51 (69) |
| SA-ECG | 13 (12) | 2 (6) | 11 (15) |
| Pathology | 8 (8) | 6 (19) | 2 (3) |
| Family history | 92 (87) | 28 (88) | 64 (86) |

*n, number; CMR, cardiac magnetic resonance; ECG, electrocardiogram; SA-ECG, signal-averaged electrocardiogram.*

** In a minority of cases, although the test was performed, one or more parameters were not reported (refer to Table 2).*
