# Supplementary material for: The arrhythmogenic cardiomyopathy phenotype associated with PKP2 c.1211dup variant
Source: Neth Heart J. 2023 Jul 28;31(7-8):315–23. doi: 10.1007/s12471-023-01791-2 (PMC10400759; doi:10.1007/s12471-023-01791-2)
Supplement: Supplementary file 5 — Table S2 Definitions [file 12471_2023_1791_MOESM5_ESM.docx]

**Table S2** Definitions

| Ventricular arrhythmia | Composite endpoint of the following events: sudden cardiac death, sudden cardiac arrest, ventricular fibrillation, spontaneous sustained ventricular tachycardia (lasting ≥30 s at ≥100 b.p.m. or with haemodynamic compromise requiring cardioversion) and appropriate implantable cardioverter defibrillator intervention |
| --- | --- |
| Sudden cardiac death | Unexpected natural death from a cardiac cause within a short time period (<1 hour) |
| Sudden cardiac arrest | loss of functional cardiac mechanical activity in association with an absence in systemic circulation, without spontaneous recovery (requiring resuscitation) |
| Ventricular fibrillation | Rapid (ventricular rate usually >300 bpm), grossly irregular electrical activity with marked variability in electrocardiographic waveform, or out-of-hospital cardiac arrest with unknown first recorded electrical activity |
| Sustained ventricular tachycardia | Tachycardia (heart rate >100 bpm) of ventricular origin (wide complex, with QRS duration >120 ms) with a duration of >30 seconds or terminated via medical intervention |
| Appropriate implantable cardioverter device intervention | Any registered implantable cardioverter defibrillator shock or anti-tachycardia pacing as a reaction to ventricular fibrillation or ventricular tachycardia |
| Cardiac syncope | A short, self-limited loss of consciousness with rapid onset most likely due to a cardiac cause without signs of reflex-mediated/neurological causes |
| Symptomatic heart failure | Heart failure as classified as New York Heart Association class 2 or higher by cardiological diagnosis |
| Arrhythmogenic cardiomyopathy diagnosis | Fulfilment of 2010 Task Force Criteria for definite arrhythmogenic right ventricular cardiomyopathy diagnosis (score 4 or higher) |
| Date of presentation | Date on which the first genetic test results letter was sent concerning successful *PKP2* testing |
| Date of end of follow-up | Date of heart transplantation or death. Alternatively, date of last appointment with a cardiologist after *PKP2* testing of which there is a medical record available (journal or letter) |
| Probands | Individuals first presenting to arrhythmogenic cardiomyopathy-related medical care for reasons other than family history |
| Family members | Individuals first presenting to arrhythmogenic cardiomyopathy-related medical care for family history |
| Endurance sport | Bethesda classification of sports class C (dynamic component >70%) |
| Hypertension | As noted in medical records, or systolic pressure >140 mmHg and/or diastolic pressure >90 mmHg and/or use of antihypertensive medicines specifically prescribed for hypertension |
| (Ex-)Smoker | History of at least 100 cigarettes or equivalent tobacco product, or active daily smoker (one or more tobacco products every day) |
| Dyslipidaemia | As noted in medical record, or total cholesterol >7 mmol/l or use of statins |
| Supraventricular tachycardia | Any electrically registered supraventricular, non-sinus tachycardia (>100 beats per minute) with the exclusion of atrioventricular nodal re-entry tachycardia |
| CMR ventricular dysfunction | Ejection fraction >1.96 standard deviations below the reference population mean |
| CMR ventricular dilatation | Indexed end-diastolic volume >1.96 standard deviations above the reference population mean |
| Echocardiographic ventricular dysfunction | Any ventricular function described as less than normal by eyeballing in echocardiographic reports |
| Echocardiographic ventricular dilatation | Any degree of ventricular dilatation described in echocardiographic reports |
| Premature ventricular contraction burden | Number of premature ventricular contractions registered per 24 hours by most recent ambulatory electrocardiogram / 100 000 (estimated daily number of contractions) |
| High premature ventricular contraction burden | Premature ventricular contraction burden >1% |
