# Supplementary material for: The arrhythmogenic cardiomyopathy phenotype associated with PKP2 c.1211dup variant
Source: Neth Heart J. 2023 Jul 28;31(7-8):315–23. doi: 10.1007/s12471-023-01791-2 (PMC10400759; doi:10.1007/s12471-023-01791-2)
Supplement: Supplementary file 6 — Table S3 Genetic sequencing techniques and genes tested [file 12471_2023_1791_MOESM6_ESM.docx]

**Table S3** Genetic sequencing techniques and genes tested

|  | **Total (n=106)** | **Probands (n=32)^*^** | **Family members**  **(n=74)** | **Males (n=51)** | **Females (n=55)** |
| --- | --- | --- | --- | --- | --- |
| **Genetic sequencing technique** | | | | | |
| Sanger sequencing | 79 | 15 | 64 | 37 | 42 |
| NGS panel^†^ | 12 | 10 | 2 | 6 | 6 |
| WES | 4 | 4 | 0 | 2 | 2 |
| MLPA/CNV detection software | 8 | 8 | 0 | 4 | 4 |
| Unknown | 12 | 4 | 8 | 6 | 6 |
| **Arrhythmogenic cardiomyopathy genes tested** | | | | | |
| PKP2 | 106 | 32 | 74 | 51 | 55 |
| DES | 16 | 14 | 2 | 9 | 7 |
| DSC2 | 29 | 27 | 2 | 17 | 12 |
| DSG2 | 32 | 26 | 6 | 20 | 12 |
| DSP | 28 | 26 | 2 | 16 | 12 |
| JUP | 28 | 26 | 2 | 16 | 12 |
| TMEM43 | 30 | 28 | 2 | 17 | 13 |
| PLN | 24 | 22 | 2 | 14 | 10 |
| FLNC | 7 | 7 | 0 | 4 | 3 |

*n, number; NGS, next generation sequencing; WES, whole exome sequencing; MLPA, multiple ligation-dependent probe amplification; CNV, copy number variant.*

** Multiple genetic sequencing techniques were employed for eight probands.*

*†NGS panels contained at least the following genes: ACTC1, ACTN2, ALPK3, BAG3, CRYAB, CSRP3, DES, DSC2, DSG2, DSP, FHL1, GLA, JPH2, JUP, LAMP2, LMNA, MIB1, MYBPC3, MYH7, MYL2, MYL3, NEXN, PKP2, PLN, PRDM16, PRKAG2, RBM20, SCN5A, TAZ, TCAP, TMEM43, TNNC1, TNNI3, TNNT2, TPM1, TTN, TTR, VCL.*
