# Supplementary material for: The arrhythmogenic cardiomyopathy phenotype associated with PKP2 c.1211dup variant
Source: Neth Heart J. 2023 Jul 28;31(7-8):315–23. doi: 10.1007/s12471-023-01791-2 (PMC10400759; doi:10.1007/s12471-023-01791-2)
Supplement: Supplementary file 7 — Table S4 Other class ≥ 3 genetic variants in PKP2 c.1211dup carriers at time of genetic testing [file 12471_2023_1791_MOESM7_ESM.docx]

**Table S4** Other class ≥ 3 genetic variants in *PKP2* c.1211dup carriers at time of genetic testing

| **Sex** | **Proband status** | **Variants** | **Classification** | **Associated phenotype** | **Diagnostic criteria fulfilled?** |
| --- | --- | --- | --- | --- | --- |
| Male | Proband | *DSG2* c.1003A>G | 3 | Arrhythmogenic cardiomyopathy | Yes (TFC 5) |
| Female | Proband | *DSG2* c.1003A>G | 3 | Arrhythmogenic cardiomyopathy | Yes (TFC 8) |
| Male | Family member | *DSG2* c.1003A>G | 3 | Arrhythmogenic cardiomyopathy | No (TFC 3) |
| Female | Family member | *DSG2* c.1003A>G | 3 | Arrhythmogenic cardiomyopathy | No (TFC 3) |
| Female | Proband | *LMNA* c.1303C>T | 3^*^ | Dilated cardiomyopathy | No |
| Female | Family member | *LMNA* c.1303C>T | 3^*^ | Dilated cardiomyopathy | LV measurements unavailable |
| Female | Family member | *LMNA* c.1303C>T | 3^*^ | Dilated cardiomyopathy | No |
| Female | Family member | *JUP* c.607C>T | 3 | Arrhythmogenic cardiomyopathy | No (TFC 3) |
| Male | Proband | *RYR2* c.6952A>G | 3 | Catecholaminergic polymorphic ventricular tachycardia | No |
| Male | Proband | *TTN* c.9839C>A c.100139T>A | 3 | Dilated cardiomyopathy | No |
| Female | Proband | *PRDM16* c.3097G>A | 3 | Dilated cardiomyopathy | LV measurements unavailable |

*Variants are classified according to the American College of Medical Genetics and Genomics and the Association for Molecular Pathologist guidelines for the interpretation of sequence variants. Class 3 denotes a variant of uncertain significance. Class 5 denotes a pathogenic variant. TFC, task force criteria; LV, left ventricle.*

** LMNA c.1303C>T has recently been reclassified from class 3 to 5 by the Netherlands cardiogenetic working group and is now regarded as pathogenic, leading to a low penetrant form of dilated cardiomyopathy.*
