# Supplementary material for: The arrhythmogenic cardiomyopathy phenotype associated with PKP2 c.1211dup variant
Source: Neth Heart J. 2023 Jul 28;31(7-8):315–23. doi: 10.1007/s12471-023-01791-2 (PMC10400759; doi:10.1007/s12471-023-01791-2)
Supplement: Supplementary file 8 — Table S5 Summary of PKP2 founder variant characteristics [file 12471_2023_1791_MOESM8_ESM.docx]

**Table S5** Summary of *PKP2* founder variant characteristics

|  | **Total (n=159)** | **c.235C>T (*n*=53)** | **c.397C>T (*n*=42)** | **c.1211dup (*n*=46)** | **c.2489+1G>A (*n*=18)** |
| --- | --- | --- | --- | --- | --- |
| **Demographics** |  |  |  |  |  |
| Proband, n (%) | 54 (34) | 16 (30) | 13 (32) | 15 (33) | 9 (50) |
| Male sex, n (%) | 86 (54) | 25 (47) | 21 (50) | 28 (60) | 11 (61) |
| Age at presentation, mean±SD, y | 38±18 | 41±20 | 40±16 | 31±16 | 36±18 |
| **VA Symptoms, n (%)** |  |  |  |  |  |
| Sudden cardiac death | 3 (2) | 2 (4) | 0 | 0 | 1 (6) |
| Sudden cardiac arrest | 8 (5) | 1 (2) | 1 (2) | 4 (9) | 2 (11) |
| VT/VF | 61 (38) | 18 (34) | 12 (29) | 20 (43) | 11 (61) |
| Appropriate ICD intervention | 17 (11) | 3 (6) | 6 (14) | 7 (15) | 1 (6) |
| VA (combined endpoint) | 63 (40) | 18 (34) | 14 (33) | 20 (43) | 11 (61) |

*Symptoms contributing to the VA combined endpoint were not mutually exclusive. As a result, the sum of symptoms exceeds the number of VA combined endpoints.*

*n, number; SD, standard deviation; y, years; VT, sustained ventricular tachycardia; VF, ventricular fibrillation; ICD, implantable cardioverter defibrillator; VA, ventricular arrhythmia.*
